# Efficient Culturing and Genetic Manipulation of Human Pluripotent Stem Cells

**DOI:** 10.1371/journal.pone.0027495

**Published:** 2011-12-15

**Authors:** Robert T. Schinzel, Tim Ahfeldt, Frank H. Lau, Youn-Kyoung Lee, Alicia Cowley, Tony Shen, Derek Peters, David H. Lum, Chad A. Cowan

**Affiliations:** 1 Department of Stem Cell and Regenerative Biology, Harvard Stem Cell Institute, Harvard University, Cambridge, Massachusetts, United States of America; 2 Center for Regenerative Medicine, Massachusetts General Hospital, Boston, Massachusetts, United States of America; 3 Cardiovascular Research Center, Massachusetts General Hospital, Boston, Massachusetts, United States of America; 4 Institut für Biologie - Mikrobiologie, Fachbereich Biologie, Chemie, Pharmazie, Freie Universität Berlin, Berlin, Germany; 5 Harvard College, Harvard University, Cambridge, Massachusetts, United States of America; 6 Huntsman Cancer Institute, University of Utah, Salt Lake City, Utah, United States of America; 7 Department of Oncological Sciences, University of Utah, Salt Lake City, Utah, United States of America; University of Southern California, United States of America

## Abstract

Human pluripotent stem cells (hPSC) hold great promise as models for understanding disease and as a source of cells for transplantation therapies. However, the lack of simple, robust and efficient culture methods remains a significant obstacle for realizing the utility of hPSCs. Here we describe a platform for the culture of hPSCs that 1) allows for dissociation and replating of single cells, 2) significantly increases viability and replating efficiency, 3) improves freeze/thaw viability 4) improves cloning efficiency and 5) colony size variation. When combined with standard methodologies for genetic manipulation, we found that the enhanced culture platform allowed for lentiviral transduction rates of up to 95% and electroporation efficiencies of up to 25%, with a significant increase in the total number of antibiotic-selected colonies for screening for homologous recombination. We further demonstrated the utility of the enhanced culture platform by successfully targeting the *ISL1* locus. We conclude that many of the difficulties associated with culturing and genetic manipulation of hPSCs can be addressed with optimized culture conditions, and we suggest that the use of the enhanced culture platform could greatly improve the ease of handling and general utility of hPSCs.

## Introduction

Since the derivation of human embryonic stem cells [1], their growth and maintenance in culture have remained challenging. When compared to mouse pluripotent stem cells (mPSCs), the human counterparts (hPSCs) are less robust, more prone to spontaneous differentiation, difficult to culture as single cells, and less amenable to genetic manipulation. With the generation of human induced pluripotent stem cells [2]–[4], there has been increased interest in the use of hPSCs for a variety of applications. Recently, the introduction of defined media conditions, feeder-free culture systems, and chemicals to facilitate survival of hPSCs as single cells [5]–[7] have led to significant improvements, yet an efficient and robust culture methodology has been lacking.

We used a combination of recently published hPSC culture protocols and their further optimization to develop a protocol that we term the enhanced culture platform (ECP). We extensively evaluated this platform and compared it to one of the more widely used culture method, here termed the standard culture platform (SCP). We developed multiple lines of evidence that culturing hPSCs using the ECP significantly facilitates their handling and genetic manipulation. Use of the ECP maintained the pluripotency and genetic integrity of hPSCs over long-term culturing and passaging. The ECP improved replating efficiencies and viability of single-cells when passaging hPSCs. This culture platform also increased the viability of hPSCS after freezing and thawing. Importantly, the ECP yielded higher clonogenic efficiency, increased transduction by lentiviral vectors, and improved electroporation efficiencies of hPSCs. Finally, we were readily able to perform homologous recombination using the ECP. Thus, the use of the ECP for growth, maintenance, and manipulation of hPSCs provides a robust and efficient culture methodology that promises to improve the utility of hPSCs.

## Results and Discussion

The ECP was the combination of a feeder free culture system utilizing Geltrex [5], TeSR defined media [7], Accutase [8] to dissociate and detach cells and Rock-Inhibitor (Y-27632) [6] to stabilize the subsequent intermediate single cell state. This culture platform was extensively evaluated and compared to the standard culture platform (SCP) of hPSCs in feeder free conditions, consisting of a combination of Geltrex, TeSR and Dispase.

In order to establish that the ECP was capable of maintaining the pluripotency and genetic integrity of hPSCs over extended culturing, we passaged HUES9 [9] and BJ-RiPSC [10] cells over 15 times using the ECP. Throughout this culture period, the cells maintained well-defined, phase-bright borders, a high nucleus-to-cytoplasma ratio, and prominent nucleoli. We further evaluated the cells immunohistochemically for markers of pluripotency including OCT4, SOX2, NANOG and TRA-1-81 (Figure S1A) and found them to be positive for each of the markers. We also confirmed high expression of two master regulators of pluripotency, *OCT4* and *NANOG*, in cells grown using the ECP by quantitative reverse transcription followed by polymerase chain reaction (qRT-PCR) amplification (Figure S1B). We also found that cells of both lines grown using the ECP for more than 15 passages formed teratomas comprising all three embryonic germ layers (Figure S1C) and remained karyotypically normal (Figure S1D).

**Figure 1:**
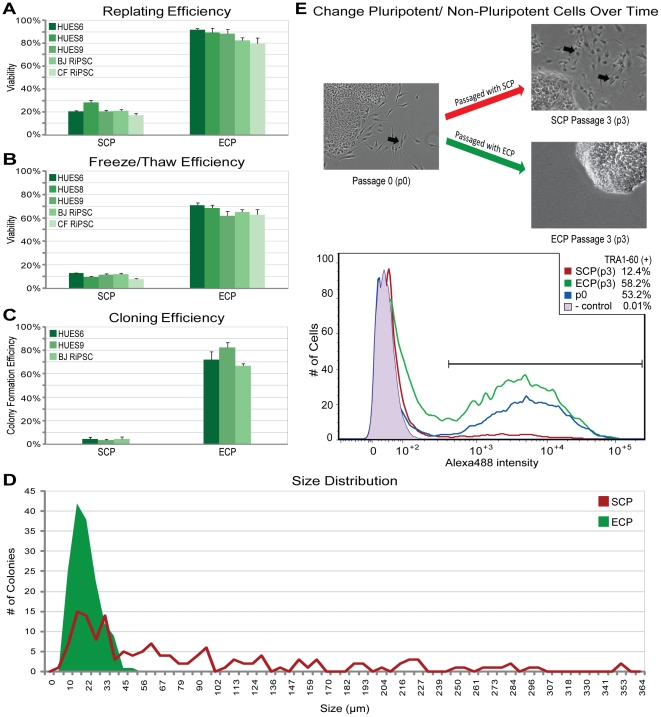
Comparing cell culture of hPSCs using the standard (SCP) or the enhanced culture platform (ECP). **A**) Quantification of cells reattached 2 h after passage in a total of 5 cell lines either using the SCP or ECP to passage the cells (n = 3 per cell line, standard error, p = 1.68^−06^). **B**) Quantification of cells reattached after being frozen in liquid nitrogen for 7–10 d and subsequently thawed (n = 3 per cell line, standard error, p = 1.69^−06^). **C**) To determine the clonogenic potential, a limiting dilution assay on a total of 3 cell lines was performed (96 cell plated per cell line, standard error, p = 0.002). Counted were only colonies with positive immunoflourescence staining for the pluripotency markers OCT3/4 and NANOG. **D**) Colony size distribution in µm, 3 days after passage with either the SCP or ECP (n = 150 colonies each). **E**) Representative images of BJ RiPSC colonies before and after being passaged 3 times with SCP or ECP respectively (black arrows indicates differentiated, non-pluripotent cells) and quantification of the change via flow cytometry utilizing TRA1-60 as a pluripotency marker.

We next compared the ECP to a well-established culture methodology (SCP) with respect to the ease of handling and maintaining hPSCs in culture. We first determined the percentage of cells reattaching after passage (often referred to as the replating efficiency) using three human embryonic stem cell (hESC) lines and two human induced pluripotent stem cell (hiPSC) lines and found a significant increase (p = 2×10^−6^) from an average 23% (SCP) to 85% (ECP) of cells that replated and remained viable (Figure 1A). As a consequence, we were able to dilute the standard split ratio of 1∶4 with the SCP to as much as a 1∶15 ratio with the ECP, allowing for more rapid expansion of hPSCs. We next sought to determine if the ECP might also improve the viability of hPSCs after freezing and thawing (Figure 1B). In all 5 hPSC lines, the percentage of viable cells recovered after freezing and thawing significantly improved (p = 2×10^−6^) from an average of 10% (SCP) to an average of 65% (ECP). The ability to quickly expand hPSCs in culture using the ECP as well as quickly create large stocks of viable frozen cells should thereby reduce the time necessary to set up experiments, as well as reduce the total time in culture for hPSCs as they are prepared for experiments.

To investigate the clonogenic potential of hPSCs grown with the ECP, the cloning efficiency in three hPSC lines was determined by limiting dilution (Figure 1C). We found that an average of 4% of plated single cells grown in SCP were able to form clonogenic colonies in comparison to almost 70% grown using the ECP (p = 0.002). As the Rho Associated Kinase (ROCK) Inhibitor (Y-27632) has been previously shown to stabilize the hESC single-cell state [6], [11] and is a component of the ECP, a series of experiments were performed to determine the effect of ROCK-Inhibitor in the ECP (Figure S2). While inclusion of ROCK-Inhibitor improved the replating, freeze/thaw, and cloning efficiency of hPSCs, most of the improvement observed with the ECP appeared to be due to the combination of the newly introduced culture techniques rather than the addition of the chemical.

With routine use of the ECP, we made two additional observations. First, dissociation of hPSCs to single cells resulted in more uniform colony sizes. We quantified the colony size distribution by measuring the surface area of 150 colonies three days after passage with either the ECP or the SCP and found a reduction in variability with the ECP (Figure 1D). Second, we observed an increase in the proportion of Tra-1-60 positive cells in culture over three passages (Figure 1E). We quantified this observation by measuring TRA-1-60 via flow cytometry using two hPSC lines that had a relatively high percentage of TRA-1-60 negative cells admixed in culture (42% in HUES1 and 47% in BJ RiPSC). After repeated passaging using the SCP, both hPSC lines showed a marked decrease in the amount of TRA1-60-positive cells [HUES1: 58% in passage 0 (p0) to 40% in passage 3 (p3); BJ RiPSC: 53% in p0 to 12% in p3]. In contrast, hPSCs cultured using the ECP increased the percentage of TRA-1-60-positive cells over time (HUES1: 58% in p0 to 66% in p3; BJ RiPSC: 53% in p0 to 58% in p3). As hPSCs are known to fluctuate in their expression of cell surface markers such as TRA-1-60, this observation may not correspond to an increase in the number of pluripotent cells when cultured with the ECP. In order to determine if the ECP does in fact reduce the proportion of non-pluripotent cells over time additional markers of pluripotency would need to be assessed over a much longer period of time in culture (i.e. 10 passages or more). The increased proportion of TRA-1-60 positive hPSCs together with the reduced colony size variation, may further reduce variability and improve standardization and reproducibility of experiments with hPSCs.

**Figure 2:**
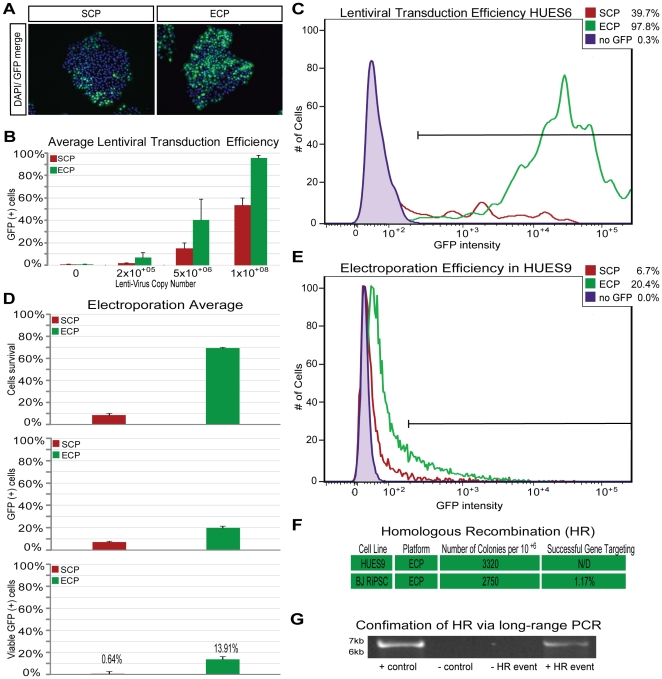
Comparing genetic manipulation efficiencies of hPSCs using either the standard (SCP) or the enhanced culture platform (ECP). **A**) Fluorescence image of Lentiviral transduction with an LV-GFP reporter indicate differences in efficiency and distribution of GFP+ cells (lentivirus copy number 5×10^+06^ per 30.000cells). **B**) Quantification of lentiviral transduction efficiency via flow cytometry towards the LV-GFP reporter with various lentivirus copy numbers (30.000 cells per setup) in 4 different cell lines (standard error, p = 0.03; cell lines: HUES6, HUES9, CF RiPSC and BJ RiPSC). **C**) Representative flow cytometry plot for HUES6 lentiviral transduction efficiency. **D**) Average efficiency of an EP-GFP reporter plasmid delivered into the HUES9 line via electroporation. The top bars indicate the amount of cells surviving the procedure, the middle bars the percentage of cells that transiently express GFP measured via flow cytometry, and the bottom bar indicate the total percentage of viable GFP positive cell in regards to the initial cell number (n = 3 per platform, standard error). **E**) Representative flow cytometry plot for HUES9 cells expressing GFP after electroporation. **F**) The table indicates the amount of colonies available for screening after a single electroporation per 10^6^ cells and subsequent antibiotic selection. **G**) Of these colonies, 85 were screened for successful gene targeting through homologous recombination via long range PCR.

We next sought to evaluate the ECP using two standard methods of genetic manipulation, lentiviral transduction and gene targeting. Lentiviral transduction has been previously reported in hPSCs using ultra-concentrated virus [12], although the reported efficiency in hPSCs remained relatively low (70%–80%) as compared to several other cell types in which transduction efficiencies of 100% can be routinely achieved without ultra-concentrating the virus. Upon transduction of hPSCs grown using the SCP with lentiviral particles designed to constitutively express green fluorescent protein (LV-GFP), GFP-positive cells were found predominantly at the borders of hPSC colonies. In contrast, hPSCs grown in the ECP and subsequently transduced with LV-GFP displayed GFP-positive cells throughout the hPSC colonies (Figure 2A). To quantify the overall efficiency of lentiviral transduction, four hPSC lines grown with either the SCP or the ECP were transduced with LV-GFP using a range of viral particle concentrations, and the percentage of GFP-positive cells was evaluated via flow cytometry (Figure 2B). A significant increase (p = 0.03) in transduction efficiency was observed in hPSCs grown with the ECP (96%) as compared to cells grown with the SCP (54%). In all of the four hPSC lines tested, the transduction efficiencies exceeded 95% with the use of ECP and without the need for ultra-concentration of the virus. The introduction of overexpression and knockdown constructs via lentivirus with greater than 95% efficiency should allow for the routine establishment of hPSC populations that homogenously express the desired factors without the need for selection strategies.

Finally, we investigated the utility of the ECP for the delivery of plasmids into hPSCs via electroporation for homologous recombination (HR). We first determined the viability of cells electroporated using either the ECP or the SCP and found a significantly increased (p = 0.002) viability of the cells electroporated with the ECP, with an average of 69% surviving the procedure (Figure 2D top). Additionally, the overall percentage of cells that transiently expressed GFP from an electroporated plasmid significantly improved (p = 0.004) from an average of 7.4% (SCP) to 20% (ECP) (Figure 2D middle), thus allowing for a more than 20-fold increase of the total number of viable GFP-positive cells from 0.64% to 13.91% of the initial number of cells. We then attempted to duplicate the previously reported targeting by HR of the *ISL1* locus [13]. After a single electroporation of 1×10^6^ cells, 3,320 colonies of HUES9 cells and 2,750 colonies of BJ RiPSC cells were obtained after antibiotic selection (Figure 2F). We evaluated 85 of the BJ RiPSC colonies for HR via long-range PCR and found one successful event (Figure 2G). The efficiency of HR at this locus with the ECP (1.17%) was comparable to what had been previously reported (1.42%). Thus, the recombination frequency at the locus does not appear to change with alteration of culture conditions, but use of the ECP allows for highly efficient target construct delivery and, given the increase in the number of colonies available for screening, should thereby facilitate gene targeting.

In conclusion, the ECP allows for dissociation and replating of single hPSCs, significantly increases viability and replating efficiency, and improves freeze/thaw viability and cloning efficiency of hPSCs. The growth of hPSCs with the ECP also reduced colony size variation and might further reduce the proportion of spontaneously arising non-pluripotent cells. When combined with standard methodologies for genetic manipulation, we found that the enhanced culture platform allowed for lentiviral transduction rates of up to 95% and electroporation efficiencies of up to 25%, with a significant increase in the total number of antibiotic-resistant colonies. Thus, the growth, maintenance, and manipulation of hPSCs with the ECP significantly facilitates the ease of utilizing hPSCs and should ultimately facilitate many applications of hPSC research.

## Methods

### Ethics statement

All animal procedures were approved by the Massachusetts General Hospital subcommittee on research animal care under animal protocols 2009N000050. Mice were maintained at a barrier facility at the Centre for Regenerative Medicine, Massachusetts General Hospital under a 12 hr light/12 hr dark cycle at constant temperature (22°C) with free access to food and water. All efforts were made to minimize suffering.

### Maintenance of pluripotent cells

hESCs and hiPSCs were cultured feeder free on Geltrex (Invitrogen) in the chemically defined medium mTESR1 (Stem Cell Technologies). ECP passage: hPSCs were dissociated with 20% Accutase (StemPro®) in PBS for 5–10 minutes at 37°C until the cells could easily be detached with pipetting. Accutase was removed and cells were replated in mTESR1 +0.4 µM ROCK-Inhibitor (Y-27632 Cayman Chemical). ROCK-inhibitor was removed 3–6 h after replating. SCP passage: hPSCs were dissociated by adding Dispase StemCell Tech, 15–20 mg/10 cm^2^ plate and were incubated at 37°C for 3–5 minutes until colony borders started to detach. After repeated washing with DMEM cells were lifted with a cell scraper and the colonies manually disrupted by pipetting several times.

### Quantifcation of the cell viability

To measure the cell viability of the replating efficiency, freeze/thawing and electroporation experiments, the cells were detached with Accutase 2–3 h after being replating. The cells were counted via Hausser Bright-Line Hemacytometer. All counting was done in triplets.

### Immunocytochemistry

The following antibodies were used: Immunostaining- α-SOX2 (Abcam ab15830, 1/200), α-TRA1-81 (Millipore, MAB4381, 1/350), NANOG (Millipore, AB9220, 1/300), OCT3/4 (Abcam, ab19857, 1/300), and Alexa Fluor secondary antibodies (Invitrogen). DAPI stain was used in a 1∶5000 dilution to mark cell nuclei. The Images were made either with a Nikon Digital Sight camera mounted to a Nikon Eclipse Ti-S microscope or a Olympus DP72 camera mounted to a Olympus 1×71 microscope. The software packages NIS-Elements and Olympus DP2-BSW were used for image analysis.

### RNA extraction, cDNA synthesis, and quantitative RT-PCR

hPSCs were lysed in buffer RLT (Qiagen) with 1% β-Mercaptoethanol and the total RNA purified via the RNeasy mini kit (Qiagen) according to the manufacturer's instructions. The RNA yield was determined using the NanoDrop ND-1000 spectrophotometer (NanoDrop Technologies) and 500 ng of total RNA was further converted to cDNA using the Superscript First-Strand Kit (Invitrogen). Quantitative RT-PCR was performed using Quantifast-SYBR Green PCR mix (Qiagen) and the Realplex Mastercycler (Eppendorf) with 25 ng of total RNA per reaction. The reaction took place in the presence of Origene STAR qPCR primer pairs against OCT3/4 (HP206340), NANOG (HP215086) or NESTIN (HP209533).

### Teratoma formation

For the teratoma assay, 5×10^6^ cells of either HUES9 or BJ RiPSC were harvested, spun down, and all excess media was removed. In a 20-week old female SCID mouse, the capsule of the right kidney was gently elevated, and one droplet of concentrated cells was inserted under the capsule. At week 6, when adequate tumor size was observed, the tumor was harvested, fixed in 4% Paraformaldehyde, run through an ethanol gradient, and stored in 70% ethanol. Specimens were sectioned and H&E staining. Slides were imaged with a Leica light microscope.

### Karyotyping

Cells were submitted to Cell Line Genetics for analysis.

### Freeze/thawing

Cells were dissociated either through Accutase digest (ECP) or by Dispase digest (SCP), transferred to mFreSR® (Stem Cell Technologies) and immediately frozen down. After 2–3weeks in liquid N2 the cells were thawed and plated on Geltrex coated plates in either mTESR1 with 0.4 µM ROCK-Inhibitor (ECP) or mTESR1 (SCP).

### Limiting dilution assay

The various cell lines were diluted to a concentration of 0.5cell/96well and two full 96well plates of each setup was prepared. After 7–10days the cells were fixated with 4% Paraformaldehyde, analyzed via Immunocytochemistry and screened for colonies expressing the pluripotency marker NANOG and OCT3/4.

### Flow cytometry analysis

Cells were detached via Accutase digestion, and fixed for 20 minutes at 4°C in 2% Paraformaldehyde. The cells which were subjected immunocytochemistry were initially counted and 1×106 cells were transferred into polypropylene tubes. Staining for the TRA1-60 antigen was performed using an antibody conjugated to alexa fluor 488 (Biolegend, 330613) in 200 µl PBS with 5% FBS. The cells were counted with a FACSCalibur flow cytometer (BD Biosciences) and the data analyzed using the FlowJo software package (Treestar).

### Colony size measurement and variability

Images of random parts of the plate were made either with a Nikon Digital Sight camera mounted to a Nikon Eclipse Ti-S microscope. Three days after replating of single cells, 20 pictures of random areas of the plates were taken and the colony sizes of 150 colonies of each platform were measured with the NIS elements BR 3.10 software.

### Production of lentivirus and transduction

We used a third-generation, Tat-free packaging system [14] to produce recombinant lentivirus. The system consists of two packaging plasmids—pMDL, pREV—, the plasmid coding for VSV-G envelope and a vector for constitutive GFP under the CMV promoter (LV-GFP). All four plasmids were transfected into HEK293FT cells using calcium chlorate as previously reported [15] and the viral supernatant was collected 48 and 72 hours later. The supernatant was filtered through a 0.45 µm filter and the RNA copy numbers per ml determined using the Lenti-X qRT-PCR titration kit. For the transduction hPSCs were dissociated via ECP/SCP and replated. Immediately after reattaching, approximately 2 h after the replating, the lentivirus was added to the plate and incubated for 2 hours at 37°C. The viral supernatant was then removed, and cells were washed with DMEM and mTESR1 was added. 48 hours after transduction the cells were collected, fixed and analyzed for GFP expression via flow cytometry.

### Electroporation

The Cells were detached with either Accutase or Dispase filtered through a 40 µm cell strainer (BD Falcon) and counted. 40 µg of linearized DNA (EP-GFP) were mixed with 3×10^6^ cells in 800 µl PBS. An electric pulse was given in a 0.4 cm cuvette (Bio-Rad Gene Pulser®) with 320 V/200 µF [16]. The cells were recovered and plated on Geltrex coated plates in TeSR and 0.4 µM/ml ROCK-Inhibitor. Viability and efficiency was determined 24 h after electroporation via cell counting and flow cytometry.

### Cell-lines

The following human embryonic stem cells lines were used for the experiments: HUES1, HUES2, HUES6, HUES8 and HUES9 all first described in Cowan et al., 2004. The following human induced pluripotent stem cell lines were used for the experiments: BJ RiPSC and CF RiPSC first described by Warren et al., 2010. The HEK293FT cell line used for Lenti-Virus production is distributed Invitrogen (R700-07).

## Supporting Information

Figure S1 **Long term culture of hPSCs with ECP dosn't effect pluripotency and Karyotype.** HUES9 and BJ RiPSC cells were cultured 15 consecutive times with the ECP and analyzed for pluripotency via **A**) Immunohistochemistry with the indicated antibodies; nuclei were visualized with DAPI stain, via **B**) quantitive reverse transcription real time PCR for OCT3/4, NANOG and the negative control NESTIN a neural progenitor cell marker (normalized to HPRT)(n = 3), via **C**) Teratomaformation of HUES9 hESCs in immunodeficient SCID mice (black arrow top left indicates neural rosette; black arrow top right - pigmented epithelia, grey arrow top right gut-like epithelia; bottom left- muscle-like tissue; black arrow bottom right cartilage). **D**) Cell Karyotype of HUES9 hESCs after 19× passages.
(TIF)

Figure S2 **The influence of ROCK-Inhibitor.** **A**) The amount of cells reattached were determined 2 h after passage in a total of 5 cell lines either using the SCP or ECP each with and without 0.4 µM of ROCK-Inhibitor (n = 3 per cell line, standard error). **B**) Quantification of cells reattached after being frozen in liquid nitrogen for 7–10 d and subsequently thawed in the presence or absence of 0.4 µM of ROCK-Inhibitor (n = 3 per cell line, standard error). **C**) Determination of the clonogenic potential, through a limiting dilution assay on a total of 3 cell lines was performed with and without the addition of 0.4 µM of ROCK-Inhibitor (96 cell plated per cell line, standard error). Counted were only colonies with positive immune-fluorescence staining for the pluripotency markers OCT3/4 and NANOG.
(TIF)
