# Supplementary figures and images for: Efficient Culturing and Genetic Manipulation of Human Pluripotent Stem Cells
Source: PLoS One. 2011 Dec 15;6(12):e27495. doi: 10.1371/journal.pone.0027495 (PMC3240614; doi:10.1371/journal.pone.0027495)

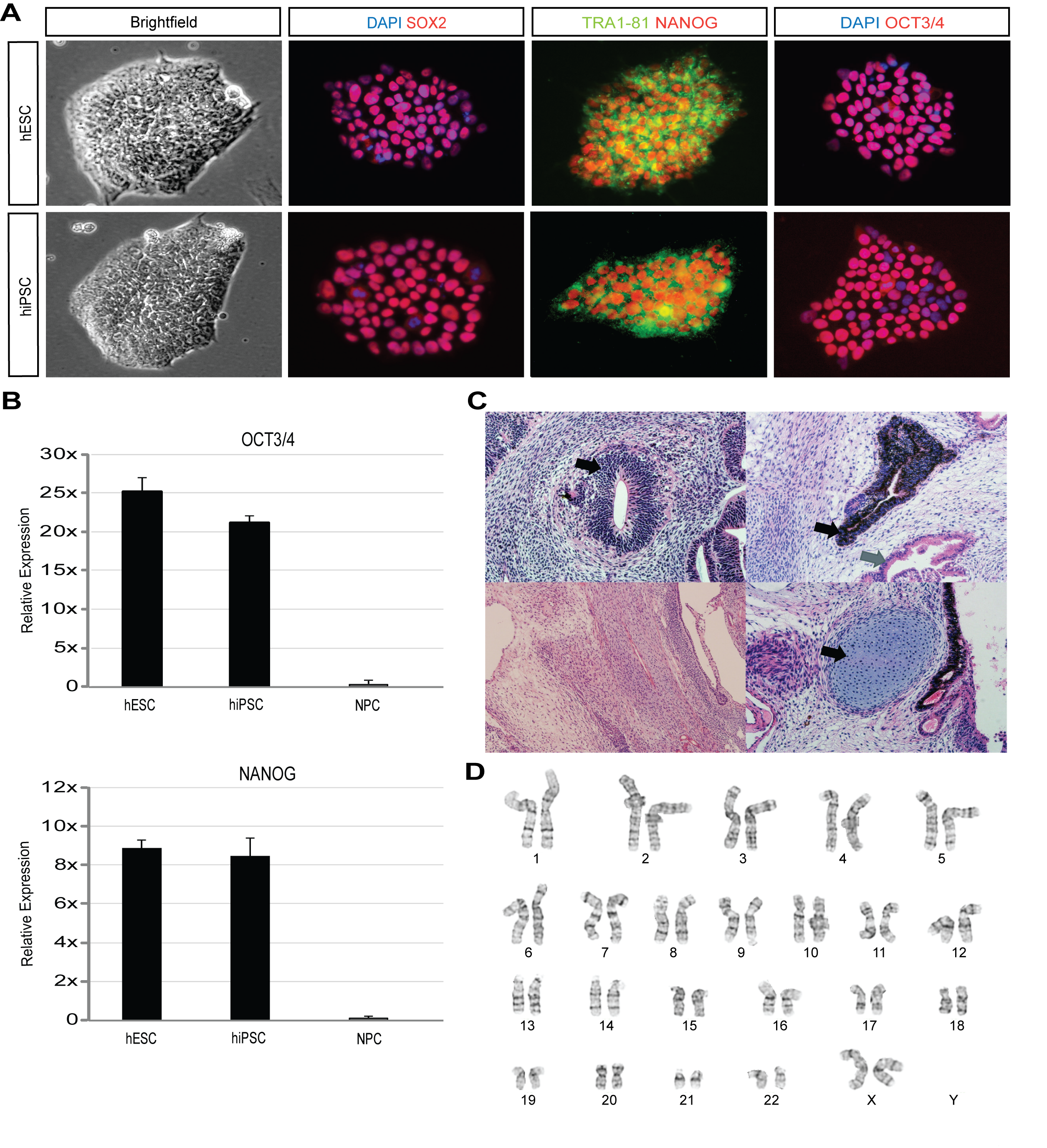

Supplement: Figure S1 — Long term culture of hPSCs with ECP dosn't effect pluripotency and Karyotype. HUES9 and BJ RiPSC cells were cultured 15 consecutive times with the ECP and analyzed for pluripotency via A) Immunohistochemistry with the indicated antibodies; nuclei were visualized with DAPI stain, via B) quantitive reverse transcription real time PCR for OCT3/4, NANOG and the negative control NESTIN a neural progenitor cell marker (normalized to HPRT)(n = 3), via C) Teratomaformation of HUES9 hESCs in immunodeficient SCID mice (black arrow top left indicates neural rosette; black arrow top right - pigmented epithelia, grey arrow top right gut-like epithelia; bottom left- muscle-like tissue; black arrow bottom right cartilage). D) Cell Karyotype of HUES9 hESCs after 19× passages. (TIF) [file pone.0027495.s001.tif]

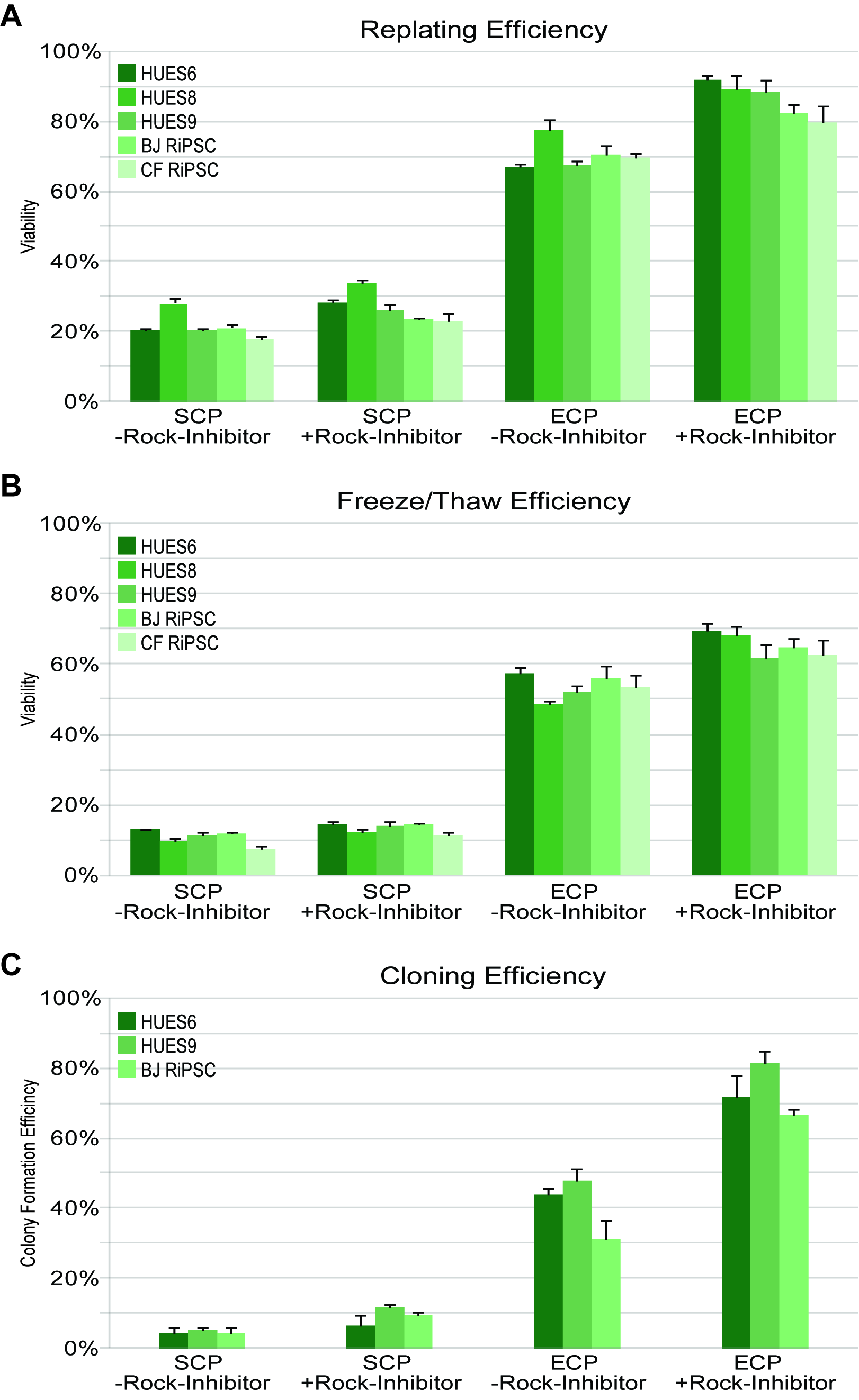

Supplement: Figure S2 — The influence of ROCK-Inhibitor. A) The amount of cells reattached were determined 2 h after passage in a total of 5 cell lines either using the SCP or ECP each with and without 0.4 µM of ROCK-Inhibitor (n = 3 per cell line, standard error). B) Quantification of cells reattached after being frozen in liquid nitrogen for 7–10 d and subsequently thawed in the presence or absence of 0.4 µM of ROCK-Inhibitor (n = 3 per cell line, standard error). C) Determination of the clonogenic potential, through a limiting dilution assay on a total of 3 cell lines was performed with and without the addition of 0.4 µM of ROCK-Inhibitor (96 cell plated per cell line, standard error). Counted were only colonies with positive immune-fluorescence staining for the pluripotency markers OCT3/4 and NANOG. (TIF) [file pone.0027495.s002.tif]
